# Modeling Neurodevelopmental and Neuropsychiatric Diseases with Astrocytes Derived from Human-Induced Pluripotent Stem Cells

**DOI:** 10.3390/ijms22041692

**Published:** 2021-02-08

**Authors:** Baiyan Ren, Anna Dunaevsky

**Affiliations:** 1 Department of Biochemistry and Molecular Biology, University of Nebraska Medical Center, Omaha, NE 68198, USA; baiyan.ren@unmc.edu; 2 Department of Neurological Science, University of Nebraska Medical Center, Omaha, NE 68198, USA

**Keywords:** astrocytes, human-induced pluripotent stem cells, neurodevelopmental disorders, neuropsychiatric diseases

## Abstract

Accumulating studies demonstrate the morphological and functional diversity of astrocytes, a subtype of glial cells in the central nervous system. Animal models are instrumental in advancing our understanding of the role of astrocytes in brain development and their contribution to neurological disease; however, substantial interspecies differences exist between rodent and human astrocytes, underscoring the importance of studying human astrocytes. Human pluripotent stem cell differentiation approaches allow the study of patient-specific astrocytes in the etiology of neurological disorders. In this review, we summarize the structural and functional properties of astrocytes, including the unique features of human astrocytes; demonstrate the necessity of the stem cell platform; and discuss how this platform has been applied to the research of neurodevelopmental and neuropsychiatric diseases.

## 1. Introduction

Cognitive, sensory, and social impairments associated with neurodevelopmental disorders, including Down syndrome, Rett syndrome, and schizophrenia, negatively impact the quality of life of affected individuals and their families. The brain, as the etiological site, is the most complex organ and remains incompletely studied. These disorders have relatively high heterogeneity in phenotypes and complexity in mechanisms, making it challenging to understand the pathogenesis and identify therapeutic targets. To this end, extensive efforts have been made to explore the pathology and potential therapy of these diseases. Previous research has mainly focused on neuronal deficits, explaining some of the significant cognitive and behavioral phenotypes observed in patients. However, the realization of the broad range of glial functions in normal development has led to exploration of the role of glial cells in disease pathogenesis.

Astrocytes, one of the most heterogeneous and abundant type of glial cells in the brain [1,2,3], possess a high degree of morphological and functional diversity [1,4,5]. Unlike neurons, astrocytes are not electrically excitable and are not thought to directly participate in information transmission. However, astrocytes regulate multiple aspects of brain function, including modulation of neuronal development [6,7,8] and synaptic activity [9], regulation of ionic homeostasis [10], recycling of neurotransmitters [11], and maintenance of the blood–brain barrier [12]. Thus, impairments in the morphology and function of astrocytes could exert a profound influence on the homeostasis of the brain, contributing to cellular mechanisms underlying the cognitive, behavioral, and affective impairments in neurodevelopmental and neuropsychiatric disorders.

Animal models, such as drosophila, zebrafish, and rodents, have been developed over decades to study the molecular and genetic mechanisms involved in the etiology of these disorders [13,14,15]. Furthermore, investigations have been performed using neuroimaging approaches on patients [16,17] and cellular analysis on human postmortem tissues [18]. However, animal models and human tissue both have limitations in that animal models have different genetic backgrounds from humans and, meanwhile, postmortem tissue is not easily accessible and does not allow discernment between causality and compensating mechanisms.

A breakthrough in biological research occurred in 2006 when Yamanaka and his colleagues successfully reprogrammed murine fibroblasts to pluripotent stem cells [19]. They soon succeeded in reproducing the protocol in human fibroblasts, generating human-induced pluripotent stem cells (hiPSCs) [20]. In the past decade, multiple protocols have been generated to differentiate hiPSCs to specialized cell types in the central nervous system (CNS) [21,22,23,24,25,26]. Among them, hiPSC-astrocytes pave the path to understanding the contribution of human astrocytes to brain disorders. Therefore, in this review, we will discuss the recent accomplishments in the differentiation of hiPSCs to astrocytes and the application of these cells to the study of neurodevelopmental and neuropsychiatric disorders, highlighting the gap in knowledge filled by human astrocyte models as well as current limitations and opportunities of these models.

## 2. Astrocyte Development and Heterogeneity

During vertebrate CNS development, neurons and astrocytes are generated from the same neural precursors: radial glial cells. Their cell bodies are retained in the ventricular zone, and bipolar processes reach the pial and ventricular surfaces [27]. Although radial glial cells transform into both neurons and astrocytes, astrogenesis is temporally separated from neurogenesis. At the end of neurogenesis and cortical development, radial glial cells undergo asymmetric division and translocate to the cortical plate, transforming into astrocytes in the subventricular zone [28].

Astrocytes that develop in different brain regions show different morphologies. While astrocytes in some brain regions, such as the cerebellum (Bergmann glia) and the retina (Müller cells), retain their radial morphology into adulthood, this morphology is lost by most astrocytes in other regions of the CNS. Four subtypes of astrocytes in the cerebral cortex have been classified: interlaminar astrocytes originating from the soma located in Layer I, protoplasmic astrocytes in Layers II-VI, varicose projection astrocytes in Layers V–VI, and fibrous astrocytes in white matter. Interlaminar and varicose projection astrocytes are exclusively found in the cortex of higher primates and humans. Protoplasmic astrocytes, the most well-studied subtype of astrocytes, have a recognizable bushy morphology, express canonical astrocytic markers such as GFAP, S-100β, and Aldh1l1 [29], and were once considered to be a generally homogenous population. However, recent transcriptomic and proteomic profiling provided evidence for substantial astrocytic diversity. Astrocytes in different brain regions, cortical layers, and within the layer exhibit transcriptomic heterogeneity [2,30,31]. In addition, careful analysis of astrocyte morphology has uncovered subpopulations of protoplasmic astrocytes with layer-specific morphological features such as branching complexity and specific interactions with synapses [30,32]. Advanced understanding of the potential association between astrocyte morphology, gene expression, and function will help to elucidate the contribution of astrocytes to neurodevelopmental and neuropsychiatric disorders.

## 3. Astrocyte Function

### 3.1. Water and Ion Homeostasis

Astrocytes play a significant role in water and ion homeostasis in the brain. Control of extracellular K^+^ concentration is one of the canonical functions of astrocytes and is achieved through K^+^ channels (predominantly Kir4.1), Na^+^/K^+^-ATPase pumps, and solute carrier transporters [33,34,35]. Following neuronal activity, excess K^+^ is taken up by astrocytes and is distributed via gap junctions. The uptake of K^+^ leads to a reduction in extracellular osmolarity, resulting in water flux through AQP4 channels co-localized with Kir4.1 on astrocyte endfeet [36]. In addition to K^+^ homeostasis, astrocytes also participate in buffering sodium and chloride ions and extruding protons from the cytoplasm [37]. Astrocytic regulation of water and ion homeostasis is critical for the normal function of neurons.

### 3.2. Calcium Signaling

Unlike neurons, astrocytes are not electrically excitable cells. They do not fire action potentials; instead they communicate in the form of intracellular calcium elevation, which either occurs spontaneously or is evoked by various stimuli, including neurotransmitters, ligands, and mechanical stimulation [38]. Astrocytes interact with synapses via thousands of fine processes [39] and interact with blood vessels via their endfeet [40]. In response to neuronal activity and cerebral blood flow, astrocytes modulate neuronal activity and energy supply via Ca^2+^ signaling and release of gliotransmitters such as glutamate, ATP, and D-serine [40,41].

An increasing number of neurotransmitters have been observed to induce Ca^2+^ signals in astrocytes, including glutamate [42], gamma-aminobutyric acid GABA [43], dopamine [44,45], and norepinephrine [46]. Ca^2+^ signals in the different compartments of astrocytes, specifically soma, branches, and microdomains, arise from different sources and exert different downstream effects [38,47]. In the soma, the endoplasmic reticulum (ER) is the main source of Ca^2+^, where it is released through activation of inositol 1,4,5-trisphosphate receptor, type 2 (IP3R2) located in the ER membrane [48]. IP3R2 signaling is either triggered by the upstream activation of G-protein-coupled receptors (GPCR) or induced by Ca^2+^-induced Ca^2+^ release (CICR) [48,49,50]. In addition, accumulating studies show the involvement of ion channels, such as plasma membrane Na^+^/Ca^2+^ exchanger [51] and transient receptor potential A1 (TRPA1) channel [52] in Ca^2+^ signals. Moreover, mitochondria are also found to participate in Ca^2+^ transients in fine processes [53].

Increases in intracellular Ca^2+^ in different cellular compartments were reported to induce astrocytic release of gliotransmitters, such as glutamate and D-serine, and modulate synaptic function (see below). In addition to its role in regulating synaptic activity, TRPA1-mediated Ca^2+^ fluctuation has also been shown to regulate the insertion of neurotransmitter transporters into the astrocytic membrane [52]. Moreover, astrocytic Ca^2+^ signals contribute to supplying energy to the brain in several ways. These include regulation of cerebral blood flow via induction of astrocytic release of arachidonic acid-derived messengers [54], insertion of glucose transporters into the astrocytic cell membrane [55], and regulation of breathing [56]. Thus, diverse sources of Ca^2+^ elevation in astrocytes exist that have essential roles in maintaining the homeostasis of the CNS under physiological conditions.

### 3.3. Synaptic Development

As the brain’s of information transmission unit, synapses undergo formation and elimination during brain development, memory formation, and learning to maintain dynamic and precise connections and normal brain function. Astrocytes interact with neurons closely in a structure termed the “tripartite synapse”, where fine processes of astrocytes partially ensheath pre- and postsynaptic structures, providing the structural basis for bidirectional communication between astrocytes and neurons [39]. The effect of astrocytes on synapse formation was first characterized in purified cultures of retinal ganglion cells (RGCs), in which RGCs developed more synapses when cultured with astrocyte feeder layers or astrocyte-conditioned media [57]. Subsequent studies identified multiple astrocyte-secreted factors, including cholesterol, thrombospondins, hevin, SPARC, and glypicans, that have discrete roles in regulating synapses [58,59,60,61,62]. These factors not only regulate structural development of synapses but also functional maturation. Functional regulation occurs presynaptically through regulation of neurotransmitter vesicle release probability and postsynaptically through regulation of the number, subunit composition, and stability of neurotransmitter receptors [63].

Astrocytes are also involved in synapse elimination. Early in brain development, neurons generate an excessive number of projections and synapses. During the critical period, some synapses are tagged and undergo extensive phagocytosis mediated by astrocytes and microglia, while the remaining synapses are stabilized and organized into the mature neural circuit [64]. Astrocytes mediate synaptic elimination directly and indirectly. Direct mechanisms involve astrocytic elimination of synapses through the MEGF10 and MERTK pathways [65]. Indirect mechanisms involve the classical complement cascade. In the retinogeniculate system, TGF-beta released by astrocytes induces the production of C1q in neurons to tag weak synapses, leading to the activation of the classical complement cascade and subsequent removal of tagged synapses by microglia [64,66].

### 3.4. Synaptic Function and Plasticity

Beyond development, astrocytes regulate synaptic function and plasticity through various mechanisms. Astrocytes can regulate synaptic transmission by rapidly removing neurotransmitters from the extracellular space on a millisecond timescale [67,68,69]. Excitatory amino acid transporters located in the astrocytic membrane, such as GLAST and GLT1, mediate the uptake of glutamate [70,71]. Glutamate is then metabolized by glutamate dehydrogenase to support energy production [72] or converted to glutamine, which is released to neurons [73]. Astrocytes also regulate the uptake of inhibitory neurotransmitters, such as GABA, by the transporter GAT3 [68].

Astrocytic release of gliotransmitters, such as glutamate, has been found to potentiate neurotransmitter release, synchronize neuronal activity, and enhance synaptic strength [74,75,76,77,78]. The release of another gliotransmitter, D-serine, also contributes to long-term synaptic potentiation and of motor skill learning [79,80,81]. Moreover, glycogenolysis in astrocytes not only generates glucose as the general energy supply to the brain but also releases lactate, which is imported into neurons during long-term memory formation [82]. Astrocytes also participate in long-term memory consolidation and allocation [83,84]. These results suggest the significance of astrocytes in synaptic refinement and learning and memory, implicating the involvement of astrocytes in the pathology of several neurodevelopmental and neuropsychiatric diseases.

## 4. Human-Specific Characteristics of Astrocytes

Although there is considerable conservation of astrocytic biology between rodents and primates, differences in the number, morphology, function, and genome of rodent and primate astrocytes are being increasingly recognized [31,85]. Compared with protoplasmic and fibrous astrocytes in rodents, corresponding astrocytes in humans have larger territories and more complex morphologies, including longer processes and a larger number of branches [32]. Due to their increased size and complexity, human astrocytes are estimated to contact 2 million synapses as compared to 20,000–120,000 in the rodent brain [32,85,86,87]. In addition, two subtypes of astrocytes identified in the human cerebral cortex are absent in rodents: the interlaminar astrocytes located near the pial surface and the varicose projection astrocytes that reside in the deep layers [32]. Human astrocytes are also functionally different from rodent astrocytes. For example, while rodent fetal astrocytes respond to glutamate, human fetal astrocytes do not. Conversely, rodent adult astrocytes are unresponsive to glutamate, while human adult astrocytes respond to glutamate [31,88]. Human astrocytes propagate calcium waves four times faster than rodent astrocytes [32]. Human astrocytes also exhibit different inflammatory responses [89]. Moreover, there are different transcriptomic profiles in human and rodent astrocytes [90]. For example, ryanodine receptor 3, responsible for CICR, and murine retrovirus integration site 1, a protein regulating intracellular calcium stores, are specifically enriched in human astrocytes [31]. This unique gene expression could contribute to the morphological and functional differences observed between human and rodent astrocytes.

The divergence in morphology, function, and gene expression among astrocytes across species indicates that caution and consideration are needed when extrapolating findings from rodents to humans. Moreover, high failure rates of drug candidates in clinical trials for neurological disorders [91,92,93,94], combined with a recognized role of astrocytes in these diseases, provide a rationale for the requirement of improved human cellular astrocyte models to better define their contribution to neurodevelopmental disorders.

## 5. Generation of hiPSC-Astrocytes

The approach of inducing pluripotent stem cells using human fibroblasts revolutionized the ability to model human disease [20,95]. Pure populations of specific cell types, such as astrocytes and neurons, can be generated or directly converted from patient fibroblast-derived iPSCs [22,23,96]. The traditional differentiation process resembles the physiological developmental stages and lineage commitment progression by exposing cells to mitogens and morphogens, mimicking in vivo developmental cues [23]. However, the direct conversion of iPSCs to astrocytes was also recently developed [97,98]. Direct conversion approaches employ overexpression of transcription factors involved in the gliogenic switch to force stem cells to enter a specific differentiation lineage within a shortened period. Both approaches have been used to model neurodevelopmental disorders [98,99].

**Figure 1:**
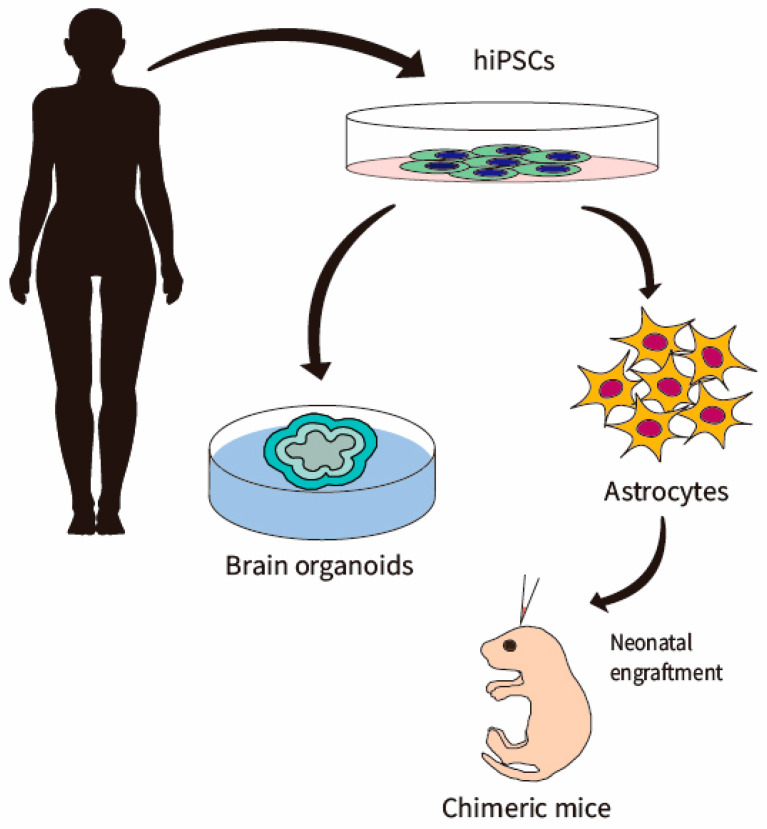
Generation of hiPSC-astrocytes. Astrocytes can be differentiated or directly converted from patient fibroblast-derived hiPSCs via 2D culture and 3D organoids. These astrocytes can be engrafted into mouse cortex to generate a chimeric mice model.

While early studies based on two-dimensional (2D) cell cultures (Figure 1) provided essential insights into brain dysfunction at the cellular level, they were limited as they did not capture the appropriate spatial organization and developmental trajectory of cells in the brain. Cerebral organoids derived from hiPSCs are self-assembled, three-dimensional (3D) aggregates with cellular diversity and cytoarchitectures resembling the human fetal brain [100,101,102,103]. Though the cerebral organoids have not completely recapitulated the gyrification, arealization, and complex neuronal circuitry of the cerebral cortex, they mimic multiple features of early human brain development in terms of lamination, cellular diversity, and gene expression. Brain region-specific organoids can even be fused to allow interactions between different brain regions [104,105,106]. Beyond what is possible in 2D culture, functional astrocytes were generated in brain organoids, accompanied by a synaptically connected neuronal network [107]. Of note, astrocytes isolated from brain organoids showed proteomic and functional similarity to human astrocytes [108].

In addition to in vitro approaches, several studies have analyzed how cells derived from hiPSCs develop in vivo. To study the properties of human astrocytes in the brain environment, investigators engrafted human glial progenitor cells (GPCs) into neonatal immunodeficient mice [109]. This chimeric mouse model helped elucidate the glial contribution to Huntington’s disease [110] and schizophrenia [111]. It was recently demonstrated that engraftment of immature astrocytes derived from hiPSCs into the mouse cortex resulted in the development of typical interlaminar astrocytes [112]. As a subtype of primate-exclusive astrocytes, the role of interlaminar astrocytes in the brain during physiological and pathological conditions has not been well studied. The chimeric mouse model presents the possibility to study how interlaminar and other astrocyte subtypes behave in the brain environment.

One difficulty in working with hiPSCs when studying neurodevelopmental and neuropsychiatric disorders is that, unlike animal models in which the genetic background of experimental and control groups is identical except for the target gene, stem cells derived from patients and healthy volunteers have extensive genomic variability, potentially masking some deficits and mechanisms. Recent application of the CRISPR/Cas9 system for editing DNA sequences of patient-derived stem cells allows for the generation of isogenic lines, circumventing the heterogeneity associated with comparing cells from patients to cells from healthy individuals. In combination with other techniques, such as genetic manipulation, single-cell transcriptome profiling, live-cell imaging, and chimeric engraftment, it is possible to study the human-specific deficits and developmental processes that are otherwise inaccessible in animal model research. Although these methods require further validation of the subtypes and developmental stages of the generated astrocytes, given the heterogeneity of human astrocytes and the lack of exact parameters to define their maturation in vitro, such studies can verify the most relevant phenotypes found in animal models and potentially identify novel human-specific phenotypes and therapeutic targets. 

## 6. Modeling Astrocytes in Neurodevelopmental Disorders

Neurodevelopmental and neuropsychiatric disorders, including intellectual disability, autism spectrum disorder (ASD), and schizophrenia, are genetically and phenotypically heterogeneous and complex. In animal models of these disorders, there has been much focus on neuronal deficits and underlying mechanisms. However, given the roles astrocytes play in the brain, it is not surprising that a growing number of studies have highlighted the contribution of astrocytes to disease pathophysiology at the molecular, cellular, and functional levels [113,114,115,116]. Here, we will describe studies that use astrocytes derived from iPSCs of patients with neurodevelopmental disorders and compare them with findings from animal models, attempting to identify the validated and novel phenotypes as well as highlight the knowledge gaps and future directions in this rapidly developing field.

### 6.1. Down Syndrome

Down syndrome (DS), or trisomy 21, has an incidence of about 1 in 700 children in the United States [117] and is the most frequent genetic cause of intellectual disability. DS results from the presence of an extra copy of human chromosome 21 (HSA21). Although DS is a neurodevelopmental disorder, many patients with DS develop Alzheimer’s disease when they reach middle-age [118].

Due to the large region of genetic homology shared by human chromosome 21 and mouse chromosome 16, several animal models of DS have been generated, including mice with full trisomy 16 (Ts16) [119], which do not survive past birth, and partial triplication (Ts65Dn). Ts65Dn mice exhibit several behavioral deficits during early postnatal development and adulthood consistent with DS patients [14,120]. The cognitive deficits could be attributed to hippocampal neuronal circuit dysfunction [121]. Similar to the abnormality in astrocytes described in both young and adult DS patients [122,123], enhanced astrogliosis was observed in Ts65Dn mice [120]. In primary astrocyte cultures from Ts65Dn mice, altered astrocyte proliferation and Ca^2+^ activity as well as reduced intracellular free zinc have been described [124,125]. Considering the limitation of animal models to recapitulate the triplication of HSA21 in DS patients, human fetal and postmortem tissues are used. Altered processing of amyloid-beta precursor protein and mitochondria dysfunction was observed in astrocytes derived from fetal DS brain [126]. In addition to the intrinsic alterations, human DS astrocytes were found to contribute to neuronal dendritic spine abnormalities [127].

To investigate the role of astrocytes in DS, astrocytes were differentiated from hiPSCs derived from DS patients [99]. Consistent with previous studies in the Ts65Dn mouse model [120,125], enhanced expression of S100B and altered Ca^2+^ fluctuations were observed in DS hiPSC-astrocytes. By treating hiPSC-neurons and neural progenitor cells (NPCs) with astrocyte-conditioned medium from DS hiPSC-astrocytes, increased neuronal apoptosis and reduced neurogenesis could be attributed to the overexpression of S100B by the DS hiPSC-astrocytes [99]. In the same study, DS hiPSC-astrocytes were transplanted into mouse brains, where reduced neurogenesis was also observed. In a similar co-culture system, neurons co-cultured with DS hiPSC-astrocytes exhibited reduced excitability, partially attributed to the elevated frequency of spontaneous Ca^2+^ fluctuations in the DS astrocytes [128]. In addition, the expression of synaptic proteins in hiPSC-neurons was reduced when co-cultured with DS hiPSC-astrocytes, supporting the idea that astrocytes contribute to the neuronal deficits in DS [129]. Gene expression studies of DS hiPSC-astrocytes revealed genome-wide transcriptional alterations, including genes involved in neuronal development and cell adhesion [99,130]. Interestingly, the differential transcriptional expression of cell adhesion-related genes was not observed in hiPSC-NPCs, indicating the emergence of these impairments during astrocytic differentiation [130]. The hiPSC-astrocyte model of DS validated astrocytic deficits described in Ts65Dn mice and patient tissues and identified several impairments in astrocyte-neuron interactions, demonstrating the contribution of astrocytes to the altered development and function of DS brain. Some of the results from different groups, however, were not consistent with each other. While elevated spontaneous Ca^2+^ fluctuations were described in DS hiPSC-astrocytes [128], a recent study with a larger number of DS hiPSC lines did not observe altered Ca^2+^ signaling in hiPSC-astrocytes [130]. Thus, it is necessary to consider the variability in the hiPSCs and astrocytes used in different labs. It is also worth noting that these results were generated from pan-astrocytes in culture without characterizing their heterogeneity and the corresponding astrocyte subtypes in the brain. These important studies demonstrated the feasibility of studying DS using the stem cell model and partially verified observations from mouse models.

### 6.2. Rett Syndrome

Rett syndrome (RTT) is an X-linked neurodevelopmental disorder that accounts for up to 10% of the cases of intellectual disability in females [131,132]. Patients with RTT exhibit regressed development in motor, verbal, and cognitive skills after reaching 6 to 18 months of age, resulting in the appearance of ASD-related symptoms. Most RTT cases are attributed to de novo mutations in the MECP2 gene, which encodes the transcriptional factor methyl-CpG-binding protein 2 (MeCP2) [133]. Mecp2-knockout mice, which have complete loss of function of MeCP2, mimic the symptoms of RTT, including delayed onset of behavioral deficits [134]. Similar to studies concentrated on other neurological diseases, initial investigations of RTT pathology focused exclusively on neurons. Astrocytes also express MeCP2 and the absence of MeCP2 in astrocytes contributes to the phenotypic regression observed in a model of RTT [135]. MeCP2-deficient astrocytes exhibited not only altered transcriptional profiles, impaired maturation, and reduced morphological complexity but also an abnormal immune response, altered regulation of glutamate homeostasis, and adverse effect on dendritic maturation [135,136,137,138,139]. In addition, the restoration of MeCP2 expression in astrocytes rescued neuronal and behavioral deficits in a mouse model, suggesting astrocytes are an integral component of the pathogenesis of RTT [140].

Although extensive studies have been performed on the RTT mouse model, Mecp2-deficient mice do not recapitulate the missense mutations in the MECP2 gene of patients with RTT, highlighting the importance of studying hiPSCs derived from RTT patients. Generating an hiPSC-based model, astrocytes were differentiated from RTT-derived hiPSCs using various culture conditions [141,142]. Neurons co-cultured with these astrocytes showed morphological and functional abnormalities, including reduced soma size, reduced length of neurites, and reduced miniature excitatory postsynaptic current frequency [141,142], consistent with the non-cell-autonomous effects of astrocytes on neuronal morphology and functions identified in previous animal studies. Furthermore, altered microtubule dynamics and vesicle transport were identified in RTT hiPSC-astrocytes, similar to observations made in Mecp2-deficient mouse astrocytes in the same study [143]. In one study using isogenic hiPSCs derived from RTT female twins, enhanced differentiation towards astrocytes was observed, likely attributed to altered astrocytic gene expression [144]. Female RTT patients are heterozygous in MECP2 mutations due to X chromosome inactivation, while the MECP2 mutations in male patients are stable, either wholly absent or mutated in all cells [145]. In a study that analyzed astrocytes derived from male RTT iPSCs, it was found that astrocyte differentiation was suppressed [146], in contrast to female patient-derived iPSCs, indicating different pathogenesis in female and male RTT patients. With the increase in the number of male RTT cases and other X-linked disorders, such as fragile X syndrome, modeling with hiPSC-astrocytes from both genders is necessary to further understand the abnormality of astrocytes, a potential cellular therapeutic target.

### 6.3. Schizophrenia

Affecting approximately 1% of the population worldwide, schizophrenia (SCZ) is a severe neuropsychiatric disorder with behavioral, cognitive, and affective impairments [147,148]. SCZ is caused by the interaction of multiple genetic and environmental factors [149]. Many animal models of SCZ have been developed, including post-weaning social isolation, phencyclidine administration, and DISC-1 mutation [15,150]. However, few animal models invariably exhibit negative and cognitive symptoms and, therefore, the mechanisms underlying disease pathology in SCZ remain poorly understood.

A glial theory of SCZ proposes that initial alterations in glial cells can lead to neuronal deficits [151,152]. Diverse astrocyte dysfunction including morphological changes, dysregulated glutamate transmission, altered inflammatory response, and oxidative stress were described [153,154], suggesting the role of astrocytes in the etiology and pathogenesis of schizophrenia. However, studies of these abnormalities in both human postmortem tissue and animal models of SCZ showed inconsistent results. Notably, increased, decreased, or unchanged GFAP expression was found in over 30 different postmortem human brain tissue studies [155]. Considering the heterogeneity of SCZ symptoms and the complexity of SCZ pathogenesis, further studies are required to clarify the astrocytic abnormalities in SCZ.

Defects in astrocytic differentiation were observed in SCZ hiPSC-derived GPCs in culture, attributed to several dysregulated genes involved in astrocytic lineage progression, including BMP signaling [156]. The same group also identified downregulated mRNA expression of potassium channels, pumps, and transporters in SCZ glia and impaired ability to regulate extracellular potassium homeostasis in SCZ astrocytes [156]. Moreover, SCZ hiPSC-astrocytes showed reduced immune response upon IL-1β exposure [157], supporting previous results which demonstrated abnormal astrocytic inflammatory pathway in SCZ human postmortem tissues [158]. To study the behavior of SCZ hiPSC-astrocytes in the brain, GPCs differentiated from SCZ patient-derived stem cells were engrafted into mice [111]. The GPCs differentiated into both oligodendrocytes and astrocytes, yielding mice chimeric for patient-derived glial cells. In chimeric mice, astrocytes differentiated from SCZ hiPSC-derived GPCs exhibited delayed astrocytic differentiation with a reduced number of GFAP-immunoreactive cells, fewer primary processes, reduced branch complexity, increased length of processes, and less coherent domain structure, suggesting non-uniform outgrowth of processes. Interestingly, these deficits were associated with behavioral impairments in the engrafted mice. In addition to the altered morphology and activity of astrocytes previously identified in postmortem human tissues and animal models of SCZ, these studies demonstrated the deficits in astrocyte development, highlighting the cell-autonomous contribution of astrocytic abnormalities to the pathology of SCZ.

## 7. Challenges and Opportunities

Among all translational models of neurodevelopmental and neuropsychiatric diseases, the human stem cell-based model serves as an irreplaceable platform due to its intimate relationship with patients. Astrocytes derived from hiPSCs with disease-specific gene mutations, therefore, will be a powerful tool to describe deficits in the genomic profile, morphology, and activity of human astrocytes and clarify the contribution of human astrocytes to the pathology of neurodevelopmental diseases. Despite the progress in modeling human neurodevelopmental disorders using hiPSCs, some challenges and limitations need to be considered.

As with other approaches, the reproducibility of results from different labs using different hiPSCs lines is critical to consider. Variability arises from multiple sources, but primarily from the varied quality of hiPSCs and different differentiation protocols. Variability can occur during the transfection of Yamanaka factors when reprogramming somatic cells into hiPSCs, resulting in intraclonal epigenetic variability. The heterogeneity between hiPSCs is also attributed to the epigenetic complexity of the patients. Many neurodevelopmental and neuropsychiatric disorders are multifactorial in origin, with genetic and environmental contributions playing critical roles in the progress and manifestation of the pathology. Other sources of variability are the different approaches used in different labs to derive astrocytes hiPSCs and the lack of a “gold standard” to characterize these hiPSC-astrocytes. They all express pan-astrocytic markers, but there is no agreement in the field about what constitutes mature astrocytes in culture. To address culture variability, future research of hiPSC-astrocytes could consider using well-validated cell lines, increasing the number of donors, and standardizing the characterization of hiPSC-differentiated astrocytes.

While the reductionist approach of studying hiPSC-differentiated astrocytes has its advantages, the resultant cells are likely have little similarity to those found in the human brain. Compared with astrocytes in the brain, which display different morphology, activity, and transcriptomic profiles in different brain regions [2], astrocytes in culture grow as a relatively homogenous population. Although some studies demonstrated regional specificity of hiPSC-astrocytes by mid/forebrain and hindbrain/spinal cord markers [23,159], most results were generated from pan-astrocytes without characterization of the heterogeneity or corresponding astrocyte subtypes. This lack of heterogeneity is partially attributed to the lack of other cell types and communication between these cell types in cultures. Another critical factor is the protracted nature of human brain development. Human adult and fetal astrocytes showed differing genomic profiles; however, considering the limited time of culturing of hiPSC-astrocytes in most studies using 2D culture, astrocyte maturation patterns might not be adequately recapitulated. The differentiation of astrocytes within brain organoids, which are typically cultured for months, can better model the maturation of hiPSC-astrocytes in vitro [160]. Long-term cultivation could also be accomplished by engrafting hiPSC-astrocytes into the mouse brain, generating cell types that are not seen in 2D cultures [112]. Both the organoid and chimera approaches have the advantage of hiPSC-differentiated astrocytes growing adjacent to and in communication with other cell types.

While hiPSC-astrocytes provide unprecedented access to elucidation of the role of genetic risk factors in early human brain development, caution is required to extrapolate the results from hiPSC-astrocytes to patients with neurodevelopmental and neuropsychiatric disorders. Despite drawbacks, patient-derived hiPSC-astrocytes provide many opportunities for the study of neurological disorders. With the development and increased access of omics techniques, such as single-cell RNA-sequencing, patient-specific genomic alterations in astrocytes can be revealed. In addition, approaches extending studies beyond cellular and molecular analyses, such as of human–mouse chimeras, will allow determining how hiPSC-astrocytes impact animal behavior relevant to the specific diseases.

## 8. Conclusions

Knowledge gained from the study of hiPSC-astrocytes derived from patients has the potential to validate previous findings from animal models and limited postmortem human tissues and identify novel therapeutic targets, thus benefiting patients and their families in the long run. While several neurodevelopmental disorders were modeled with hiPSC-astrocytes, many properties of astrocytes remain to be characterized. Combined with recent technological advances such as genomic editing, high-throughput single-cell transcriptomics, methods to study epigenetics, brain organoids, and chimeric mouse models, hiPSC-derived astrocytes have revolutionized our toolbox for modeling human disorders, especially those with complex genetic origins that are challenging to model in animals. Though these approaches require further improvements and modifications, we expect the use of hiPSC-astrocytes to significantly improve our understanding and treatment of additional neurodevelopmental and neuropsychiatric disorders.
